# Uterine and Rectosigmoid Perforation Complicated With Bilateral Tubo-ovarian Abscesses Due to Internationally Performed Dilation and Curettage

**DOI:** 10.7759/cureus.97576

**Published:** 2025-11-23

**Authors:** Alejandro H Arzola, Joseph Russell, Michael R Gonzalez Ramos

**Affiliations:** 1 Emergency Medicine, Herbert Wertheim College of Medicine, Miami, USA; 2 Surgery, Herbert Wertheim College of Medicine, Miami, USA; 3 General Surgery, Baptist Hospital of Miami, Miami, USA

**Keywords:** dilation and curettage, obstetrics and gynecology, surgical complication, traumatic bowel perforation, tubo-ovarian abscess

## Abstract

Dilation and curettage (D&C) is a common gynecological procedure performed for various indications and is generally considered safe when conducted in appropriate clinical settings. However, when performed in resource-limited environments or under non-standardized protocols, the risk of severe complications increases significantly. We report the case of a 37-year-old woman who presented with flank pain, fever, and dysuria and was found to have bilateral complex tubo-ovarian abscesses and bowel perforation following an elective D&C abortion performed abroad. This report discusses the patient’s initial presentation, diagnostic workup, and surgical management, emphasizing the importance of a multidisciplinary approach in caring for patients with complex, life-threatening complications.

## Introduction

Dilation and curettage (D&C) is a common gynecological procedure performed for diagnostic purposes, miscarriage management, or elective abortion. When conducted according to clinical guidelines established by the American College of Obstetricians and Gynecologists (ACOG), the procedure is generally considered safe, with complications such as infection, excessive bleeding, or uterine perforation occurring in less than 1% of cases [1]. However, when D&C procedures fall below accepted standards of care, the risk of serious complications increases, including uterine perforation, abscess formation, sepsis, and injuries to adjacent organs such as the bowel and bladder [2].

Before 2022, it was estimated that more than 26 million safe abortions and approximately 25 million unsafe abortions were performed annually worldwide [3]. A global review by Sedgh et al. in 2008, analyzing abortion outcomes between 1995 and 2003, found that while induced abortions occurred at similar rates in both developed and developing countries, 48% were considered unsafe, with 97% of these unsafe procedures taking place in developing nations [4]. By 2008, one in five pregnancies worldwide resulted in abortion [4].

Globally, second-trimester abortions account for 10%-15% of all abortions each year. Of those occurring at gestational ages ≥14 weeks, 92% are surgical [3]. In contrast, approximately 40% of abortions before nine weeks of gestation are medically induced [3]. Mortality rates for safe abortions are reported to be below 0.2%, whereas unsafe abortions carry a mortality rate ranging from 4.7% to 13.2% [5]. The gap between these outcomes is exemplified by a 2012 retrospective study from Tanzania that examined bowel perforation complications following illegally induced abortions at a tertiary hospital [5]. Of all patients identified as having undergone an illegal D&C for pregnancy termination, 4.2% (68 patients) experienced bowel perforation. The women ranged in age from 14 to 45 years (median: 21 years), and seven of those 68 patients (approximately 10%) died as a result of their complications [5].

This report describes a case of a 37-year-old woman who presented to the emergency department with bilateral tubo-ovarian abscesses, rectosigmoid perforation, and uterine injury following a D&C procedure performed internationally for pregnancy termination. This case highlights the importance of maintaining a high index of clinical suspicion, timely surgical intervention, and flexible management strategies when addressing severe complications of D&C procedures. The report has been prepared in accordance with the SCARE Criteria for surgical case reports [6].

## Case presentation

Here, we present the case of a 37-year-old woman, gravida 3 para 2-0-1-2, with no significant past medical history, who immigrated to the United States from Cuba one month prior to presentation. She arrived at the emergency department with chief complaints of flank pain, dysuria, and fever for one week. On obtaining a detailed history, the patient reported undergoing a D&C abortion for a six-week pregnancy in Cuba eight weeks prior to presentation. During follow-up visits in Cuba, she was noted to have either blood clots or retained products. A pelvic ultrasound (US) performed at that time reportedly showed no significant pathology. The patient stated she had not resumed menstruation for four weeks following the D&C, equating to a three-month missed cycle by the time of presentation in the United States. She denied pelvic pain or urinary symptoms after a second follow-up in Cuba. However, one week before presenting to the emergency department, she developed pelvic discomfort, flank pain, fever, and dysuria. She denied vaginal spotting and reported no sexual activity since the D&C. Review of systems was otherwise unremarkable.

On presentation, her vital signs showed a temperature of 39.4 °C, blood pressure of 110/63 mmHg, heart rate of 128 beats per minute, respiratory rate of 18 breaths per minute, and oxygen saturation of 100% on room air. Physical examination revealed normal external genitalia and no discomfort with speculum insertion. Scant bloody mucus was observed in the vaginal canal, and the cervical os was closed. Cervical motion tenderness was elicited on bimanual examination. The abdomen was slightly distended with mild diffuse tenderness, most pronounced in the lower quadrants, without signs of peritonitis. Initial laboratory evaluation demonstrated a quantitative β-hCG level of 68 U/mL (normal: 0-3 U/mL), white blood cell (WBC) count of 14,320/µL (89.6% neutrophils), hemoglobin of 8.3 g/dL, and hematocrit of 26.1% (Table 1). Transvaginal and abdominal/pelvic Doppler ultrasonography showed no evidence of intrauterine gestation or ovarian torsion but revealed a complex right ovarian cyst versus abscess measuring 4.7 × 5.4 × 4.1 cm. Endometrial thickness measured 8.9 mm.

**Table 1:** Laboratory results from the emergency room workup of the patient. ALT: alanine transaminase, AST: aspartate transaminase.

Lab	Value	Reference value
β-hCG	68 U/mL	<1 U/mL
Leukocytes	14,320/µL	4,000-11,000/µL
Neutrophils	89.60%	50%-70%
Lymphocytes	5.70%	30%-45%
Platelets	375,000/µL	150,000-450,000/µL
Hemoglobin	8.3 g/dL	12-16 g/dL
Hematocrit	26.20%	37%-47%
Sodium	136 mEq/L	136-145 mEq/L
Bicarbonate	27 mEq/L	23-28 mEq/L
ALT	22 U/L	10-40 U/L
AST	18 U/L	10-40 U/L
Globulin	4.5 g/dL	2-3.5 g/dL
Blood urea nitrogen	5 mg/dL	8-20 mg/dL
Creatinine	0.71 mg/dL	0.50-1.10 mg/dL
Lactic acid	0.9 mmol/L	0.7-2.1 mmol/L
Calcium	8.7 mg/dL	8.6-10.2 mg/dL

The patient was initially managed with an intravenous (IV) fluid bolus of 1.5 L normal saline, 800 mg ibuprofen for pain and fever control, and IV clindamycin 900 mg for preliminary antibiotic coverage. Blood cultures later revealed growth of *Peptoniphilus asaccharolyticus* and Gram-positive cocci in clusters. The patient was admitted to the medical observation floor for further management, where the antibiotic regimen was transitioned to piperacillin-tazobactam 3.375 g IV every six hours for seven days. The preliminary diagnoses upon admission included tubo-ovarian abscess, pelvic inflammatory disease, and sepsis. Despite broad-spectrum antibiotic therapy, the patient’s leukocytosis and fever persisted, prompting a change in antibiotics to cefoxitin and vancomycin. After two days of this regimen, her WBC count remained elevated at 17 K/µL.

**Figure 1:**
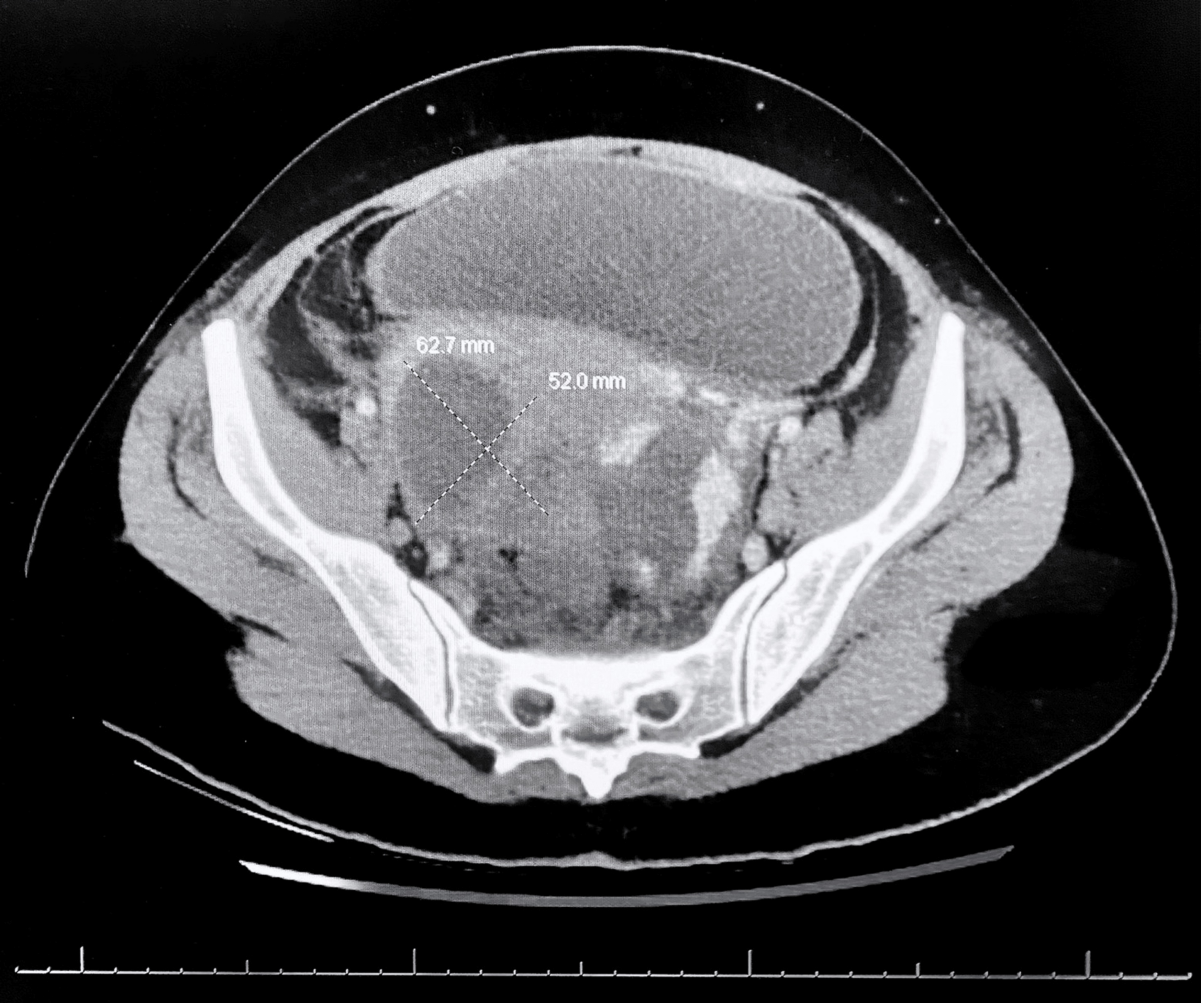
Axial contrast-enhanced CT scan of the pelvis revealing a large, irregular fluid collection measuring 62.7 mm x 52.0 mm, with adjacent inflammatory stranding, suggestive of a tubo-ovarian abscess and possible bowel perforation.

**Figure 2:**
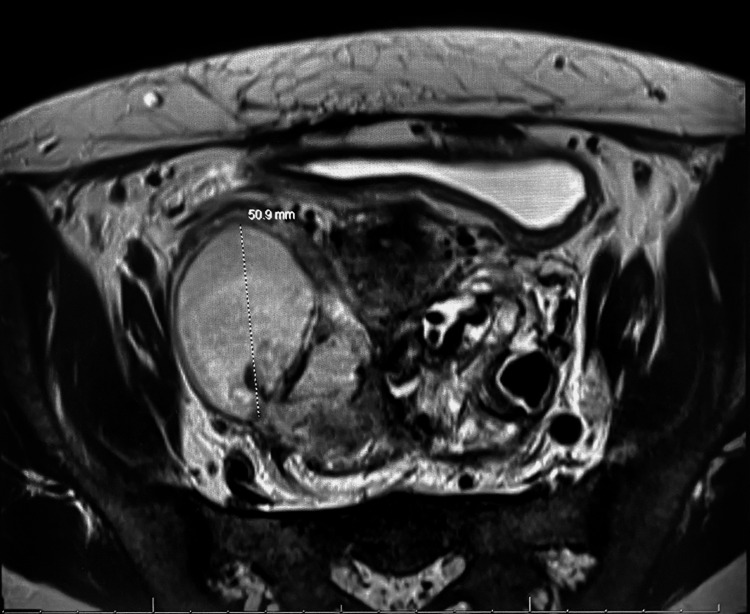
T2-weighted axial MRI of the pelvis demonstrating a large complex adnexal fluid collection measuring approximately 50.9 mm, notable inflammatory changes, and adjacent bowel loops suggest secondary involvement.

Following a multidisciplinary discussion, an abdominopelvic computed tomography (CT) scan was obtained to further evaluate the persistent sepsis and intra-abdominal pathology, given the absence of any evidence of a viable intrauterine pregnancy. The CT revealed bilateral complex adnexal cystic lesions consistent with tubo-ovarian abscesses, measuring up to 6.3 cm on the right, with likely extension along the sigmoid colonic wall, and a 4.8 cm left adnexal fluid collection surrounded by large vascular collaterals (Figure 1). The extensive collateral vasculature prompted further evaluation with magnetic resonance imaging (MRI) with and without contrast, which demonstrated that the collaterals surrounding the left adnexa originated from both the uterine and gonadal vessels, draining into the left gonadal vein (Figure 2). Given the high risk of bleeding, interventional radiology was consulted for possible minimally invasive percutaneous drainage of the abscesses. However, no safe window for drainage was identified, and the procedure was aborted. The decision was then made to proceed with robotic-assisted diagnostic laparoscopy for direct assessment and drainage of the tubo-ovarian abscesses, as well as D&C to address any potential retained products of conception within the uterus.

Initial intraoperative findings revealed extensive omental adhesions to the anterior abdominal wall and dense fibrosis between the uterus and bilateral adnexa. Both adnexa contained complex tubo-ovarian abscesses with friable, bleeding tissue involving the upper rectum, consistent with chronic perforation. Dissection was technically challenging due to the severe inflammatory process, allowing visualization only of the anterior uterine wall, uterine fundus, bilateral round ligaments, and proximal segments of the fallopian tubes. The ovaries were not identifiable, and the pelvic cul-de-sac was completely obliterated. Despite extensive hydrodissection and blunt dissection with graspers to delineate the anatomy, the dense adhesions between the sigmoid colon, rectum, and uterus prevented adequate visualization of the surgical field. Consequently, an intraoperative consultation with general surgery was requested.

Upon further exploration, profuse spontaneous bleeding from the left ovarian artery occurred, resulting in loss of visualization. Attempts at cauterization were unsuccessful, and a temporary tamponade was applied while the procedure was converted to an open laparotomy. Upon entering the peritoneal cavity, extensive friable ovarian tissue was noted, densely adherent to the rectosigmoid colon and uterus, with copious purulent material present. After drainage of the purulent contents, a chronic, contained sigmoid perforation was identified. Given the extent of the injury, a partial proctocolectomy with end colostomy creation was performed. A 15-round Jackson-Pratt (JP) drain was placed in the left lower abdomen to facilitate pelvic drainage. Pathologic examination of the resected colon and rectum demonstrated exuberant serosal adhesions, fibrinopurulent exudate, and focal transmural acute inflammation consistent with perforation. The appendix exhibited acute periappendicitis with serosal adhesions. Histopathologic findings included chronic endometritis and adnexal tissue showing acute inflammation, hemorrhage, and immature chorionic villi consistent with retained products of conception. Analysis of the pelvic abscess fluid revealed organizing hemorrhage containing immature chorionic villi and products of conception. Cultures from the tubo-ovarian abscess grew *Enterobacter cloacae*/*asburiae* and *Citrobacter braakii*.

Following the procedure, vancomycin was discontinued on postoperative day (POD) 2, and cefoxitin was discontinued at discharge. Once the patient was ambulating independently with occupational and physical therapy support, demonstrated acceptable laboratory values, tolerated a regular diet, and had adequate colostomy function, she was discharged home on POD 6. Post-discharge recovery instructions included avoiding strenuous physical activity, maintaining the regular diet established during hospitalization, and attending follow-up visits at two weeks and two months. A colostomy reversal was planned for six months postoperatively. The patient adhered to all follow-up appointments, with the JP drain removed at the two-week visit and colostomy reversal performed at six months. Following the robotic colostomy reversal, she was hospitalized for two days and discharged without complications. She was subsequently evaluated at two weeks and two months post-reversal, demonstrating full recovery without gastrointestinal or genitourinary complications.

## Discussion

Induced surgical abortions, such as D&C, are more commonly performed during the second trimester and beyond, whereas medication-induced abortions and vacuum aspirations are primarily used in the first trimester [3,7]. The D&C procedure removes products of conception more effectively than medication-induced abortions, such as misoprostol-based methods, particularly in second-trimester cases [3]. However, surgical interventions carry a higher risk, though still rare, of internal complications, which can result from factors such as improper cervical dilation technique or limited surgical experience [3]. Globally, over 90% of pregnancy terminations performed during or after the second trimester are mechanically induced through D&C [3]. Regarding vacuum aspiration, the Society of Family Planning notes that there are no significant differences between manual and electric vacuum aspiration methods in terms of completion rates, complication rates, or patient satisfaction [7]. The only notable distinction lies in procedure time, with electric vacuum aspiration being completed more quickly [7].

Uterine perforation, though rare, is a serious complication of D&C that can, in uncommon instances, lead to life-threatening sequelae. Hefler et al. conducted a retrospective review of 5,359 D&C procedures and reported 50 cases of uterine perforation (0.9%), with the uterine fundus being the most frequently affected site [8]. Such injuries may result in severe consequences, including large or small bowel perforation, ectopic pregnancy, peritonitis, hemorrhage, and abscess formation involving adjacent organs or tissues [9]. Mabula et al. examined 68 cases of illegally induced abortions in a Tanzanian tertiary hospital, 50 of which involved D&C procedures [5]. Studies like this highlight the disparities faced by healthcare systems in resource-limited regions, where available data often underrepresent the true burden of unsafe abortions due to limited reporting and publication. Mabula et al. found that the ileum (51.5%) and sigmoid colon (22.1%) were the most common sites of bowel perforation following induced abortions, with an overall complication rate of 47%, nearly half of all cases analyzed [5]. In settings with limited access to advanced imaging and timely surgical intervention, these complications may carry a high risk of mortality. Moreover, cultural and systemic barriers can delay patients’ presentation to healthcare facilities, exacerbating the severity and outcomes of such complications.

This case report describes a 37-year-old woman who underwent a D&C in her native country and narrowly avoided fatal complications from multiple tubo-ovarian abscesses and perforations of the uterus and colon. The heightened clinical suspicion of the treating physicians allowed for timely recognition of the severity of her condition and decisive intraoperative management. However, as demonstrated by this patient’s presentation, earlier intervention might have prevented the progression and severity of these complications. Preventive measures could have included regular post-procedure follow-up with serial imaging, monitoring of laboratory values such as β-hCG, and, importantly, continuity of care with a consistent healthcare provider. In this case, the patient received her initial care abroad with limited and unclear follow-up, and the details of those visits were unavailable. Consequently, proactive prevention of these complications would have been challenging.

The strengths of this case report include a detailed account of the patient’s clinical course and the procedural steps leading to the eventual exploratory laparotomy. However, a notable limitation is the lack of accessible medical history regarding the D&C procedure and follow-up visits while the patient was in Cuba. Additional information on the original procedure, post-procedural imaging, and early follow-up assessments could have provided the treating physicians in the United States with a clearer understanding of the patient’s condition. Although earlier surgical intervention during her US presentation could be considered, the complications identified, such as abscess formation and the development of collateral vasculature, appeared chronic in nature and likely evolved during the two months following the initial procedure abroad. Furthermore, the timing of her presentation to the emergency department ultimately depended on her own decision to seek care for her worsening symptoms.

This case underscores the importance of maintaining a broad differential diagnosis and exercising clinical judgment when evaluating patients presenting with atypical symptoms after procedures performed abroad. While D&C is generally considered a safe procedure, rare but serious complications can occur, particularly when follow-up care is inconsistent or performed in resource-limited settings. For clinicians, it is essential to inquire about where and how prior procedures were performed, as variations in imaging availability, surgical expertise, and postoperative care can significantly affect patient outcomes. Beyond the clinical aspects, attention to social and systemic factors, such as financial constraints, insurance status, and access to local healthcare, can help identify barriers to follow-up and inform patient counseling. Ultimately, this case highlights the dual importance of clinical vigilance and awareness of the broader context in which patients make healthcare decisions.

## Conclusions

D&C resulting in uterine perforation is a rare but serious complication that highlights the critical importance of proper surgical technique and access to appropriate medical equipment and technology. In medically underserved regions, the absence of these resources places patients at greater risk for severe outcomes, including uterine and bowel perforation, tubo-ovarian abscess, hemorrhage, and ectopic pregnancy.

This case underscores the importance of obtaining a detailed patient history, conducting a comprehensive diagnostic workup, and employing a multidisciplinary approach to effectively manage complex complications. By emphasizing these principles, clinicians can deliver timely, effective care and help prevent similar adverse outcomes. Furthermore, this case reinforces the ongoing need for global standardization of surgical practices and expanded access to safe, high-quality medical care to reduce preventable complications.
